# Roles of plant metal tolerance proteins (MTP) in metal storage and potential use in biofortification strategies

**DOI:** 10.3389/fpls.2013.00144

**Published:** 2013-05-14

**Authors:** Felipe K. Ricachenevsky, Paloma K. Menguer, Raul A. Sperotto, Lorraine E. Williams, Janette P. Fett

**Affiliations:** ^1^Centro de Biotecnologia, Universidade Federal do Rio Grande do SulPorto Alegre, Brazil; ^2^Departamento de Botânica, Universidade Federal do Rio Grande do SulPorto Alegre, Brazil; ^3^Centro de Ciências Biológicas e da Saúde, Programa de Pós-Graduação em Biotecnologia (PPGBiotec), Centro Universitário UNIVATESLajeado, Brazil; ^4^Centre for Biological Science, University of SouthamptonSouthampton, UK

**Keywords:** biofortification, iron, metal storage, manganese, MTP proteins, zinc

## Abstract

Zinc (Zn) is an essential micronutrient for plants, playing catalytic or structural roles in enzymes, transcription factors, ribosomes, and membranes. In humans, Zn deficiency is the second most common mineral nutritional disorder, affecting around 30% of the world's population. People living in poverty usually have diets based on milled cereals, which contain low Zn concentrations. Biofortification of crops is an attractive cost-effective solution for low mineral dietary intake. In order to increase the amounts of bioavailable Zn in crop edible portions, it is necessary to understand how plants take up, distribute, and store Zn within their tissues, as well as to characterize potential candidate genes for biotechnological manipulation. The metal tolerance proteins (MTP) were described as metal efflux transporters from the cytoplasm, transporting mainly Zn^2+^ but also Mn^2+^, Fe^2+^, Cd^2+^, Co^2+^, and Ni^2+^. Substrate specificity appears to be conserved in phylogenetically related proteins. MTPs characterized so far in plants have a role in general Zn homeostasis and tolerance to Zn excess; in tolerance to excess Mn and also in the response to iron (Fe) deficiency. More recently, the first MTPs in crop species have been functionally characterized. In Zn hyperaccumulator plants, the MTP1 protein is related to hypertolerance to elevated Zn concentrations. Here, we review the current knowledge on this protein family, as well as biochemical functions and physiological roles of MTP transporters in Zn hyperaccumulators and non-accumulators. The potential applications of MTP transporters in biofortification efforts are discussed.

## Introduction

Metal micronutrients such as zinc (Zn), manganese (Mn), and iron (Fe) are essential for plant metabolism and development. These micronutrients serve as substrates for a range of membrane transporters in plants, including the metal tolerance proteins (MTPs). Zn is a cofactor for more than 300 enzymes and 2000 transcription factors, being associated with auxin metabolism, maintenance of membrane integrity and reproduction (Marschner, 1995; Barker and Pilbeam, 2007; Prasad, 2012). Mn activates enzymes involved in DNA synthesis, antioxidant defense, sugar metabolism, and protein modification, and is important for photosynthesis by catalyzing water oxidation in photosystem II (Marschner, 1995; Crowley et al., 2000; Merchant and Sawaya, 2005; Williams and Pittman, 2010). Fe is necessary for redox reactions in chloroplast and mitochondria, chlorophyll synthesis, nitrogen fixation, and DNA replication (Marschner, 1995; Briat et al., 2007). As deficiency of these metals is limiting for growth, plants have developed specific acquisition and transport mechanisms for each of them (for reviews see Puig and Peñarrubia, 2009; Williams and Pittman, 2010; Conte and Walker, 2011; Hindt and Guerinot, 2012; Sinclair and Krämer, 2012).

However, metals can be harmful when in excess. Mn and Fe toxicity are two of the main limiting factors for agriculture in acid soils, which cover 30% of the planet, whereas Zn toxicity is observed in contaminated soils around mining sites (von Uexküll and Mutert, 1995; Krämer, 2010). Excess of both Zn and Mn impairs growth and leads to chlorosis, competing with other ions for binding sites (e.g., leading to Fe deficiency). Fe participates in Fenton chemistry, generating toxic levels of reactive oxygen species when in excess (Marschner, 1995; Clemens, 2001; Schutzendubel and Polle, 2002; Briat et al., 2007; Williams and Pittman, 2010; Shanmugam et al., 2011). Moreover, the Fe transporter IRT1 non-specifically transports Mn and Zn into the root symplast, as well as non-essential but toxic cations such as cadmium (Cd) and nickel (Ni), increasing their concentrations in the plant during the Fe-deficiency response (Eide et al., 1996; Korshunova et al., 1999; Baxter et al., 2008). To avoid cellular damage, heavy metals are generally chelated by low molecular weight compounds, sequestered into organelles or expelled to the extracellular space by specific transporters. One class of metal transporters involved in these functions is the Cation Diffusion Facilitator (CDF) family, also known as the Metal Tolerance Proteins (MTPs) in plants.

CDF transporters are phylogenetically ubiquitous, spanning the Archaea, Eubacteria, and Eukaryote kingdoms (Nies and Silver, 1995). CDF proteins seem to act as Metal^2+^ (Me^2+^)/H^+^ antiporters, and contain amino and carboxy cytoplasmic termini, as well as six transmembrane domains (TMD), with a few exceptions (Guffanti et al., 2002; Chao and Fu, 2004; Grass et al., 2005; Kawachi et al., 2008). The three dimensional structure of the CDF protein from *Escherichia coli* YiiP is known (Lu and Fu, 2007). The protein structure, together with other pieces of evidence, suggests that CDF transporters act as homodimers (Blaudez et al., 2003; Wei and Fu, 2006; Lu and Fu, 2007). Transported substrates include a wide range of divalent cations, such as cobalt (Co^2+^), Ni^2+^, Mn^2+^, Cd^2+^, Fe^2+^, and Zn^2+^, the latter being the most commonly transported ion (Anton et al., 1999; Persans et al., 2001; Delhaize et al., 2003; Munkelt et al., 2004; Grass et al., 2005; Montanini et al., 2007). CDF transporters are found in different membranes, such as the bacterial cell membrane, Golgi apparatus of mammals and plants, and vacuolar membranes of yeast and plants, acting in metal efflux from the cytoplasm, either to the extracellular space or into organelles (Haney et al., 2005; Peiter et al., 2007). Only one case of a CDF protein that transports Zn into the cytoplasm was described (Cragg et al., 2002).

Comprehensive phylogenetic analyses of the CDF family in several species, including bacteria, fungi, plants, and mammals, have grouped its members into three major clusters, named Zn-CDFs, Fe/Zn-CDFs, and Mn-CDFs, based on the hypothesized or confirmed transported substrate of a few members (Montanini et al., 2007). A CDF signature was found in a region that encompasses TMD II, TMD III, and the cytoplasmic loop between them. Based on multiple protein alignments and putative substrate specificity, it was also proposed that two amino acid motifs located at TMD II and TMD V are responsible for metal selectivity (Montanini et al., 2007).

**Figure 1:**
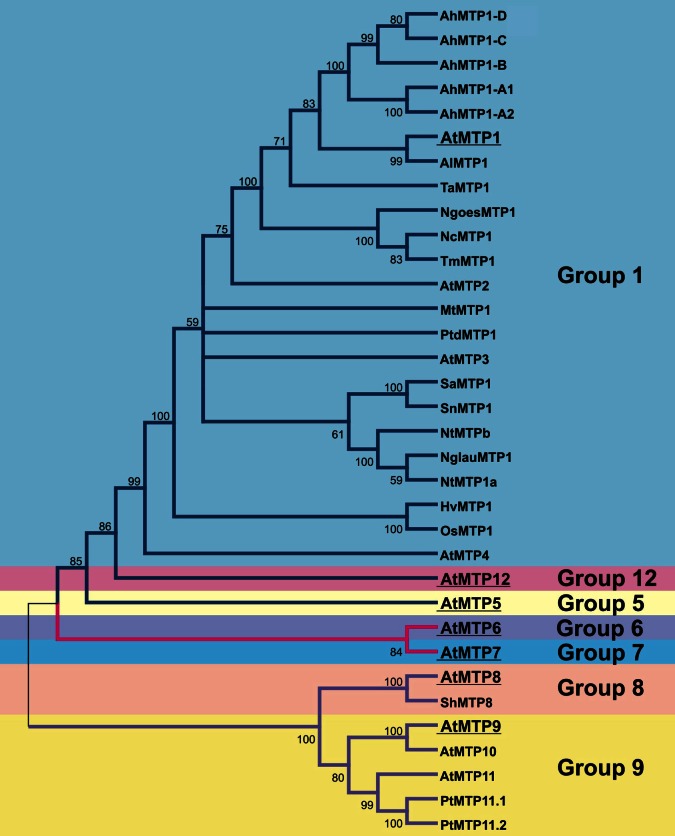
**Guide tree showing the phylogenetic groups of MTP proteins in plants.** The tree was constructed using all sequences cited in Table 1 plus all MTP proteins from *A. thaliana*, as each group of MTP was named after its first *A. thaliana* member. Founding members of each group are highlighted. Colors of branches are in blue for Zn-CDF group proteins; pink for Fe/Zn-CDF proteins; and purple for Mn-CDF proteins. The purpose of the tree is to help understand the discussion of MTP proteins presented in this review. For evolutionary relatedness analyses, we refer to the more complete datasets and trees showed by Gustin et al. (2011). For clarity, both branched and group colors are similar to those used by Gustin et al. (2011). Our phylogenetic analysis was performed using protein sequences aligned by ClustalW; the tree constructed using Neighbor-Joining algorithm, with the following parameters: pairwise deletion, Poisson correction, and 1000 replications for bootstrap confidence level estimation. Accession numbers are: from Phytozome (http://www.phytozome.org): AtMTP1(At2g46800), AtMTP2(At3g61940), AtMTP3(At3g58810), AtMTP4(At2g29410), AtMTP5(At3g12100), AtMTP6(At2g47830), AtMTP7(At1g51610), AtMTP8(At3g58060), AtMTP9(At1g79520), AtMTP10(At1g16310), AtMTP11(At2g39450), AtMTP12(At2g04620), AlMTP1(483845), OsMTP1(LOC_Os05g03780); from Genbank (http://www.ncbi.nlm.nih.gov/genbank/): AhMTP1-A2(AJ556183), AhMTP1-B(FN386317), AhMTP1-C(FN386316), AhMTP1-D(FN386315), HvMTP1(AM286795), MtMTP1(FJ389717), NcMTP1(AF275750), NglauMTP1(AB201239), NtMTP1a(AB201240), NtMTPb(AB201241), NgoesMTP1(AY044452), PtMTP11.1(EF453693), PtMTP11.2(EF453694), PtdMTP1(AY450453), SaMTP1(JF794551), ShMTP8(AY181256), SnMTP1(JF794552), TaMTP1(AY483145), TmMTP1(AY483144). Gene names are according to Table 1.

In plants, the CDFs are referred to as MTP, mainly due to their role in sequestration of excessive Zn in the vacuoles of both metal non-hyperaccumulators (e.g. *Arabidopsis thaliana*) and hyperaccumulators (e.g. *Arabidopsis halleri* and *Noccaea caerulescens*). Plant-focused phylogenetic analysis confirmed the three specificity-based clusters previously proposed (Montanini et al., 2007; Migeon et al., 2010; Gustin et al., 2011). The plant MTPs divide further phylogenetically into seven groups, and group naming follows nomenclature of MTP sequences from *A. thaliana* (Gustin et al., 2011). Sequences of all seven groups are found in each plant genome analyzed, indicating that expansion of the MTP family predates the emergence of land plants. Groups 1, 12, and 5 are part of the Zn-CDFs; groups 6 and 7, of the Fe/Zn-CDF; and groups 8 and 9, of the Mn-CDF (Gustin et al., 2011). To date, the only characterized proteins in plants are members of group 1, which includes the Zn vacuolar transporters AtMTP1 and AtMTP3 (Kobae et al., 2004; Desbrosses-Fonrouge et al., 2005; Arrivault et al., 2006); and from group 9, which includes the trans-Golgi/pre-vacuolar compartment-localized AtMTP11 (Delhaize et al., 2007; Peiter et al., 2007). The most updated and comprehensive phylogenetic tree of plant MTP proteins can be seen in Gustin et al. (2011). A simplified version of that tree (with only MTP sequences from *A. thaliana*, which are used to name MTP groups, plus sequences from proteins mentioned in this review) is shown in Figure 1, to help the reader comprehend the relationships among the different groups mentioned in the text.

Here, we review the current knowledge on MTP proteins and their roles in plant metal homeostasis, as well as studies on molecular determinants of specificity in the MTP family. We also discuss the function that MTPs play in metal hypertolerant/hyperaccumulator species. As MTP proteins have the ability to isolate metals inside cells of specific plant organs in a safe way, we discuss the potential for using these proteins in biofortification applications. The current information available on characterized plant MTP proteins as well as mutations (including single and multiple residue deletions and substitutions) that could change substrate specificity are presented in Tables 1 and 2, respectively.

**Table 1:** **Functionally characterized plant MTP proteins**.

**Protein**	**Species**	**Substrate**	**Substrate test**	**Transcriptional regulation**	**Subcellular localization**	**References**
AhMTP1-A1	*Arabidopsis halleri*	Zn	*zrc1cot1*	++Zn (↑roots)^b^	Tonoplast	Dräger et al., 2004; Shahzad et al., 2010
AhMTP1-A2	*Arabidopsis halleri*	Zn	*zrc1cot1*	++Zn (↑roots)^b^	–	Shahzad et al., 2010
AhMTP1-B1	*Arabidopsis halleri*	Zn	*zrc1cot1*	–	–	Shahzad et al., 2010
AhMTP1-C	*Arabidopsis halleri*	Zn	*zrc1cot1*	++Zn (↓shoots, ↓roots)	–	Shahzad et al., 2010
AhMTP1-D	*Arabidopsis halleri*	Zn	*zrc1cot1*	++Zn (↓shoots, ↓roots)	–	Shahzad et al., 2010
AlMTP1	Arabidopsis lyrata	Zn	*zrc1cot1*	–	–	Kim et al., 2004
AtMTP1	*Arabidopsis thaliana*	Zn	*zrc1cot1, E. coli* proteoliposomes, *X. laevis* oocytes	–	Tonoplast	Bloss et al., 2002; Kobae et al., 2004; Desbrosses-Fonrouge et al., 2005; Kawachi et al., 2008
AtMTP3	*Arabidopsis thaliana*	Zn, Co	*zrc1cot1*	−Fe, ++Zn, ++Co, ++Mn (↑roots)	Tonoplast	Arrivault et al., 2006
AtMTP11	*Arabidopsis thaliana*	Mn, Cu	INVSc2, *pmr1*	–	Pre-vacuolar compartment/trans-Golgi	Delhaize et al., 2007; Peiter et al., 2007
HvMTP1	*Hordeum vulgare*	Zn, Co	*zrc1cot1, zrc1, cot1*	–	Tonoplast	Podar et al., 2012
MtMTP1	*Medicago truncatula*	Zn	*zrc1, cot1*	++Zn	Tonoplast	Chen et al., 2009
NcMTP1	*Noccaea caerulescens*	Zn	*zrc1cot1*	–	–	Assunção et al., 2001; Kim et al., 2004
NgoesMTP1^a^	*Noccaea goesingense*	Zn, Cd, Co and Ni	*zrc1cot1, cot1, zcr1, pep5*	–	Tonoplast^c^	Persans et al., 2001; Kim et al., 2004; Gustin et al., 2009
NglauMTP1	*Nicotiana glauca*	Zn, Co	*zrc1, cot1*	–	Tonoplast^d^	Shingu et al., 2005
NtMTP1-A	*Nicotiana tabacum*	Zn, Co	*zrc1, cot1*	–	Tonoplast^d^	Shingu et al., 2005
NtMTP1-B	*Nicotiana tabacum*	Zn, Co	*zrc1, cot1*	–	Tonoplast^d^	Shingu et al., 2005
OsMTP1	*Oryza sativa*	Zn, Co, Ni, Cd, and Fe	*cot1, zrc1 cot1, smf1*, DY1455, *ycf1, ccc1, pmr1*	++Zn, ++Cd, ++Fe, ++Cu (↑shoots, ↑roots)	plasma membrane, Tonoplast^c^^,^^e^	Lan et al., 2012; Yuan et al., 2012; Menguer et al., 2013
PtMTP11.1	*Populus trichocarpa*	Mn	*pmr1*	–	trans-Golgi	Peiter et al., 2007
PtMTP11.2	*Populus trichocarpa*	Mn	*pmr1*	–	trans-Golgi	Peiter et al., 2007
PtdMTP1	*Populus trichocarpa × Populus deltoides*	Zn	*zrc1, cot1*	–	Tonoplast	Blaudez et al., 2003; Montanini et al., 2007
SaMTP1	*Sedum alfredii*	Zn	*zrc1*	–	Tonoplast	Zhang et al., 2011
ShMTP8	*Stylosanthes hamata*	Mn, Cu	INVSc2, *cnb1, pmr1*	–	Tonoplast, Endoplasmic Reticulum	Delhaize et al., 2003, 2007
SnMTP1	*Sedum aldredii*	Zn	*zrc1*	–	–	Zhang et al., 2011
TaMTP1	*Thlaspi arvense*	Zn	*zrc1cot1*	–	–	Kim et al., 2004
TmMTP1	*Thlaspi montanum*	Zn	*zrc1cot1*	–	–	Kim et al., 2004

Table shows protein transported substrate, used method for substrate determination, transcript regulation (up ↑ *or down* ↓) by metal deficiency (−) or excess (++) and tested subcellular localization.

a All three allelic variants/distinct loci (Kim et al., 2004).

b Not distinguishable by PCR, same pair of primers.

c Subcellular localization in yeast.

d First shown to be localized also in the plasma membrane by Kim et al., 2004.

e Subcellular localization in Arabidopsis thaliana.

**Table 2:** **MTP1 mutations already published are reported with the corresponding topological position and yeast complementation assays performed for different metals**.

**Protein**	**Species**	**Amino acid changes**	**Location**	**Zn**	**Co**	**Cd**	**Fe**	**Mn**	**References**
HvMTP1	*Hordeum vulgare*	WT		+	+	−	−	−	Podar et al., 2012
		Substitution Hv/At-His-loop	His-loop	+	−	×	×	×	ǁ
		Substitution Hv/At-N-His-loop	His-loop	+	−	×	×	×	ǁ
		Substitution Hv/At-C-His-loop	His-loop	+	+	×	×	×	ǁ
		Deletion VTVTT	His-loop	+	−	×	×	×	ǁ
		Substitution VTVTT/NESDD	His-loop	+	+	×	×	×	ǁ
		N206V	His-loop	+	+	×	×	×	ǁ
		S207T	His-loop	+	+	×	×	×	ǁ
		E208V	His-loop	+	+	×	×	×	ǁ
		D209T	His-loop	+	+	×	×	×	ǁ
		D210T	His-loop	+	+	×	×	×	ǁ
AtMTP1	*Arabidopsis thaliana*	WT		+	−	−	−	−	Kawachi et al., 2008
		Deletion His-loop	His-loop	+	+	−	×	×	ǁ
		Substitution At/Hv-His-loop	His-loop	+	+	×	×	×	Podar et al., 2012
		Substitution At/Hv-N-His-loop	His-loop	+	+	×	×	×	ǁ
		Substitution At/Hv-C-His-loop	His-loop	+	−	×	×	×	ǁ
		Deletion NSEDD	His-loop	+	+	×	×	×	ǁ
		Substitution NESDD/VTVTT	His-loop	+	+	×	×	×	ǁ
		V205N	His-loop	+	+	×	×	×	ǁ
		T206S	His-loop	+	−	×	×	×	ǁ
		V207E	His-loop	+	+	×	×	×	ǁ
		T208D	His-loop	+	+	×	×	×	ǁ
		T209D	His-loop	+	+	×	×	×	ǁ
		H196Q	His-loop	+	+	×	×	×	ǁ
		H201L	His-loop	+	+	×	×	×	ǁ
		H206A	His-loop	+	+	×	×	×	ǁ
		T208A	His-loop	+	+	×	×	×	ǁ
		H212N	His-loop	+	+	×	×	×	ǁ
		H212L	His-loop	+	+	×	×	×	ǁ
		C31A	N-terminal	±	−	×	×	×	Kawachi et al., 2012
		C31D	N-terminal	±	−	×	×	×	ǁ
		C31E	N-terminal	±	−	×	×	×	ǁ
		C31S	N-terminal	±	−	×	×	×	ǁ
		C36A	N-terminal	±	−	×	×	×	ǁ
		C36D	N-terminal	+	−	×	×	×	ǁ
		C36E	N-terminal	±	−	×	×	×	ǁ
		C36M	N-terminal	±	−	×	×	×	ǁ
		Deletion 2-12	N-terminal	+	+	+	×	×	ǁ
		Deletion 2–28	N-terminal	+	+	+	×	×	ǁ
		Deletion 2–55	N-terminal	−	−	−	×	×	ǁ
		C59A	TMD I	+	−	×	×	×	ǁ
		C65A	TMD I	+	−	×	×	×	ǁ
		E72A	TMD I	±	−	×	×	×	ǁ
		S81A	EL1	±	−	×	×	×	ǁ
		T86A	EL1	+	+	+	×	×	ǁ
		D87A	EL1	±	−	×	×	×	ǁ
		H90A	TMD II	−	−	×	×	×	ǁ
		L91M	TMD II	+	+	−	×	×	ǁ
		S93A	TMD II	+	−	×	×	×	Podar et al., 2012
		S93T	TMD II	+	−	×	×	×	ǁ
		D94A	TMD II	−	−	×	×	×	Kawachi et al., 2012
		A99T	TMD II	+	+	×	×	×	Podar et al., 2012
		A99V	TMD II	+	+	×	×	×	ǁ
		S101A	TMD II	+	+	+	×	×	Kawachi et al., 2012
		S104A	TMD II	+	−	×	×	×	ǁ
		T113A	IL1	±	−	×	×	×	ǁ
		T117C	IL1	−	−	×	×	×	ǁ
		F120A	IL1	+	−	×	×	×	ǁ
		R122A	TMD III	−	−	×	×	×	ǁ
		E124A	TMD III	−	−	×	×	×	ǁ
		V130A	TMD III	+	+	×	×	+	Podar et al., 2012
		I135F	TMD III	±	+	×	×	+	ǁ
		I135V	TMD III	+	−	×	×	−	ǁ
		I135G	TMD III	+	+	×	×	−	ǁ
		I135L	TMD III	+	+	×	×	+	ǁ
		I135Y	TMD III	±	+	×	×	+	ǁ
		I135Q	TMD III	+	+	×	×	+	ǁ
		I135N	TMD III	±	+	×	×	+	ǁ
		I135E	TMD III	±	+	×	×	+	Podar et al., 2012
		G140A	TMD III	+	+	×	×	+	ǁ
		Y144C	TMD III	+	+	+	×	×	Kawachi et al., 2012
		E145G	TMD III	+	+	×	×	+	Podar et al., 2012
		E145N	TMD III	+	+	×	×	+	ǁ
		E145A	TMD III	+	+	+	×	×	Kawachi et al., 2012
		R149C	EL2	+	+	+	×	×	ǁ
		E153A	EL2	+	−	×	×	×	ǁ
		E156A	EL2	+	−	×	×	×	ǁ
		N158A	EL2	±	−	×	×	×	ǁ
		N173A	TMD IV	±	−	×	×	×	ǁ
		N258A	TMD V	+	+	+	×	×	ǁ
		H265A	TMD V	±	−	×	×	×	ǁ
		D269C	TMD V	±	−	×	×	×	ǁ
		E288A	EL3	+	−	×	×	×	ǁ
		W289A	EL3	+	−	×	×	×	ǁ
		D289A	EL3	−	−	×	×	×	ǁ
		C296A	TMD VI	+	−	×	×	×	ǁ
		L298A	TMD VI	+	+	+	×	×	ǁ
		L305A	TMD VI	±	+	−	×	×	ǁ
		I312A	TMD VI	+	+	+	×	×	ǁ
		L319A	TMD VI	±	−	−	×	×	ǁ
		L298A/L305A	TMD VI	±	+	+	×	×	ǁ
		I312A/L319A	TMD VI	±	−	−	×	×	ǁ
		L298A/L305A/I312A	TMD VI	±	−	+	×	×	ǁ
		L298A/L305A/I312A/L319A	TMD VI	±	+	−	×	×	ǁ
		T323C	C-terminal	+	+	+	×	×	ǁ
		H346A	C-terminal	−	−	×	×	×	ǁ
		E347A	C-terminal	−	−	×	×	×	ǁ
		C362A	C-terminal	+	−	×	×	×	ǁ
		D373A	C-terminal	+	−	×	×	×	ǁ
		H391C	C-terminal	±	−	×	×	×	ǁ
		Q395A	C-terminal	−	−	×	×	×	ǁ
		E397A	C–terminal	±	−	×	×	×	ǁ
PtdMTP1	*Populus trichocarpa × Populus deltoides*	WT		+	−	−	×	−	Blaudez et al., 2003
		C30S	N-terminal	±	×	×	×	×	Montanini et al., 2007
		C35S	N-terminal	±	×	×	×	×	ǁ
		C64S	TMD I	±	×	×	×	×	ǁ
		D86A	TMD II	−	×	×	×	×	Blaudez et al., 2003; Montanini et al., 2007
		H89A	TMD II	−	×	×	×	×	Blaudez et al., 2003
		H89K	TMD II	−	×	×	×	×	ǁ
		D93A	TMD II	−	×	×	×	×	Blaudez et al., 2003; Montanini et al., 2007
		H260D	TMD V	−	×	×	×	×	Montanini et al., 2007
		D264A	TMD V	−	×	×	×	×	ǁ
		D264E	TMD V	−	×	×	×	×	ǁ
		D288A	TMD VI	−	×	×	×	×	ǁ
		D288E	TMD VI	−	×	×	×	×	ǁ
		L293A	TMD VI	+	×	×	×	×	Blaudez et al., 2003
		L300A	TMD VI	±	×	×	×	×	ǁ
		L307A	TMD VI	−	×	×	×	×	ǁ
		L314A	TMD VI	−	×	×	×	×	ǁ
		L293A/L300A	TMD VI	+	×	×	×	×	ǁ
		L293A/L307A	TMD VI	±	×	×	×	×	ǁ
		L293A/L314A	TMD VI	−	×	×	×	×	ǁ
		L300A/L307A	TMD VI	−	×	×	×	×	ǁ
		L300A/L314A	TMD VI	−	×	×	×	×	ǁ
		L307A/L314A	TMD VI	−	×	×	×	×	ǁ
OsMTP1	*Oryza sativa*	WT		+	+	+	+	−	Menguer et al., 2013
		L82F	EL1	±	+	+	+	+	ǁ
		L82S	EL1	±	±	+	±	−	ǁ
		H90D	TMD II	−	+	+	+	−	ǁ
		G127S	TMD III	±	+	+	+	−	ǁ
		E145G	TMD III	±	+	+	+	−	ǁ
		R149G	TMD III	+	+	+	+	−	ǁ
		L317A	TMD VI	±	+	+	+	−	ǁ

Mutations presented are substitutions, deletions or site-directed mutations.

WT, wild type; TMD, transmembrane domain; His-loop, histidine loop; IL, intracytosolic loop; EL, extracytosolic loop; +, growth in yeast complementation assay; −, no growth in yeast complementation assay; ±, partial growth in yeast complementation assay; X, not tested; ǁ, same reference.

## MTPs from Group 1 of the Zn-CDF cluster

### Group 1 MTP proteins are vacuolar Zn transporters

Group 1 of MTPs went through some duplication events during evolution, and include *MTP1*, *MTP2* (*MTP1* and *MTP2* cluster is referred as *MTP1/2*), *MTP3*, and *MTP4* sequences (Gustin et al., 2011). The *Sorghum bicolor* and *Oryza sativa* genomes lack MTP4 sequences, suggesting that the monocot lineage may have lost this gene. Phylogenetic analysis indicates that MTP1/2 and MTP3 sequences share a common ancestor; at the time of duplication, after the monocot/eudicot divergence, MTP1/2 and MTP3 most likely shared identical redundant function (Gustin et al., 2011). Although MTP1/2 and MTP3 functions diverged in *A. thaliana* (Figure 1), *Sorghum bicolor* and *Oryza sativa* MTP1 may retain the full function of the ancestral gene, as these species do not show *MTP2* and *MTP3* sequence in their genomes (Gustin et al., 2011). Transcriptional evidence suggests that genes from Group 1 are largely expressed in a variety of plants and algae, indicating the group's general importance in the photosynthetic lineage (Gustin et al., 2011).

**Figure 2:**
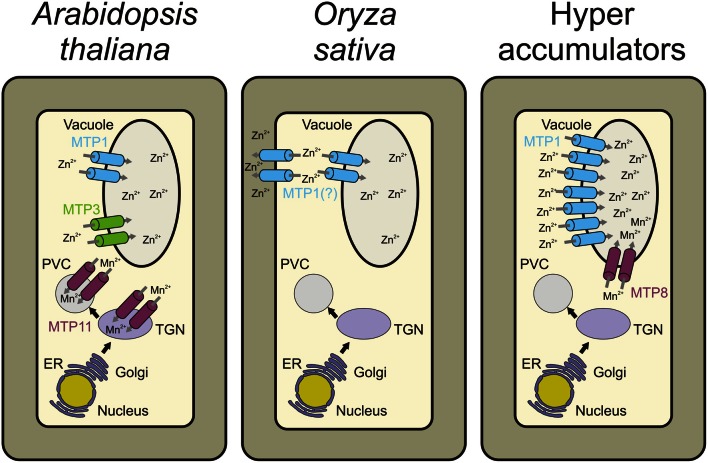
**Schematic representation of a cell and the different MTP roles in cellular metal homeostasis in each plant species.** In *A. thaliana*, MTP1 and MTP3 are vacuolar Zn transporters. Whereas MTP1 is more widely expressed in the plant, MTP3 expression is restricted to root epidermis and cortex. MTP11 transports Mn into the trans-Golgi network (TGN) and/or the prevacuolar compartment (PVC; Delhaize et al., 2007; Peiter et al., 2007). In *Oryza sativa*, MTP1 is described as a plasma membrane Zn transporter based on onion epidermal cell transient expression or as tonoplast-localized when heterologously expressed in yeast and Arabidopsis (Menguer et al., 2013). In Zn hyperaccumulators such as *A. halleri*, *N. caerulescens*, and *N. goesingense*, the MTP1 protein is highly expressed, being necessary for hypertolerance to high metal concentrations. In the Mn hyperaccumulator *S. hamata*, MTP8 transports Mn into the vacuole. Contrary to MTP1, the role of MTP8 in hyperaccumulation/hypertolerance seems to be more related to higher transport efficiency than to increased copy number (Delhaize et al., 2007).

*MTP1* and *MTP3* DNA sequences from *A. thaliana* share 68% sequence identity, and proteins have similar predicted secondary structure (van der Zaal et al., 1999; Kobae et al., 2004; Desbrosses-Fonrouge et al., 2005; Arrivault et al., 2006). Several studies have demonstrated that AtMTP1 has Zn transport activity (Table 1). When heterologously expressed in *E. coli* and reconstituted into proteoliposomes, uptake of Zn did not require a proton gradient across the liposomal membrane. More Zn accumulates in *Xenopus laevis* oocytes expressing *AtMTP1* than in water-injected oocytes. The Zn-hypersensitive yeast mutant *zrc1cot1* expressing AtMTP1 is tolerant to excessive Zn (Table 1). Similarly, AtMTP3 is able to transport Zn and Co when heterologously expressed in the *zrc1cot1* yeast mutant (Table 1). Both proteins have a function in metal detoxification by transport into the vacuole (Figure 2; Kobae et al., 2004; Desbrosses-Fonrouge et al., 2005; Arrivault et al., 2006; Kawachi et al., 2009).

Silencing of *AtMTP3* or *AtMTP1* and the disruption of *AtMTP1* by T-DNA insertion (Kobae et al., 2004; Desbrosses-Fonrouge et al., 2005; Kawachi et al., 2009) led to Zn sensitivity, which suggested that the silenced or mutant plants cannot compensate the lack of one gene by expressing the other one, and therefore *AtMTP3* and *AtMTP1* have non-redundant functions in Zn tolerance (Arrivault et al., 2006). Promoter activity of *AtMTP3* and *AtMTP1* partially overlaps in roots: the former has low but inducible expression, mainly localized to the epidermis and cortex of the root hair zone, while the later has constitutive expression, localized at the meristematic and elongation zones. *AtMTP3* expression is undetectable in shoots, whereas *AtMTP1* is expressed in young leaves (Desbrosses-Fonrouge et al., 2005; Arrivault et al., 2006). *AtMTP1* transcripts are not regulated by Zn supply, whereas AtMTP3 is up-regulated by Zn, Mn, and Co excess, as well as Fe deficiency (Bloss et al., 2002; Kobae et al., 2004; Desbrosses-Fonrouge et al., 2005; Arrivault et al., 2006; Kawachi et al., 2008). It was proposed that the AtMTP1 functions in sequestering Zn in sensitive, dividing and expanding tissues, and generating Zn stores in specific tissues of the shoot, while AtMTP3 would be involved in removing Zn from the root-to-shoot translocation pathway under high Zn influx (Sinclair and Krämer, 2012).

The MTP1 protein from the legume model plant *Medicago truncatula* can complement the Zn-susceptible *zrc1cot1* yeast double mutant (Table 1). The expression of *MtMTP1* is detected in all vegetative organs with the highest level of expression observed in leaves. Zn supplementation reduces *MtMTP1* expression in roots and increases it in stems (Table 1), while no obvious changes are detected in leaves (Chen et al., 2009). However, the subcellular localization of MtMTP1 is not known. *PtdMTP1* from a hybrid poplar (*Populus trichocarpa* × *Populus deltoides*) is constitutively expressed, complements both yeast single mutants *zrc1* and *cot1* and is localized at the vacuole (Table 1). When over-expressed in *Arabidopsis*, *PtdMTP1* confers enhanced Zn tolerance (Blaudez et al., 2003). Taken together, these results indicate that MtMTP1 and PtdMTP1 have a similar role to AtMTP1/AtMTP3 from *A. thaliana*.

The rice *OsMTP1* gene was recently characterized. Located on chromosome 5, it is most highly expressed in mature leaves and stems. *OsMTP1* expression is significantly induced by exposure to metals such as Zn, Cd, Cu, and Fe (Table 1; Lan et al., 2012; Yuan et al., 2012). Both overexpression and RNAi-mediated silencing suggest a role for the transporter in Zn, Cd, and Ni movement. *OsMTP1* expression increased tolerance to Zn, Cd, and Ni in *cot1*, *ycf1*, and *smf1* yeast mutants, respectively, during the exponential growth phase, but was not able to complement the *pmr1* mutant yeast strain hypersensitivity to Mn (Table 1; Yuan et al., 2012). More recently, heterologous expression of *OsMTP1* in the yeast strain *zrc1cot1* complemented the Zn-hypersensitivity of this mutant. OsMTP1 could also alleviate to some extent the Co sensitivity of *zrc1cot1* and rescued Fe and Cd hypersensitivity in *ccc1* and *ycf1* mutants, respectively, when tested at low concentrations of corresponding metals (Menguer et al., 2013). OsMTP1 did not complement the *pmr1* mutant for Mn transport, as reported previously. Overall, the results suggest that OsMTP1 transports Zn but also Co, Fe, Cd, and Ni, possibly with lower affinity (Menguer et al., 2013). Based on QTLs localization, *OsMTP1* was identified as a high priority candidate gene for enhancement of Fe and Zn concentrations in seeds (Anuradha et al., 2012).

There is controversy over OsMTP1 localization, reported at the plasma membrane when expressed in onion epidermal cells (Yuan et al., 2012) or at the vacuole when expressed in *Saccharomyces cerevisiae* and in the *A. thaliana* mutant *mtp1-1* (Table 1; Lan et al., 2012; Menguer et al., 2013). The barley ortholog HvMTP1 exhibits selectivity for both Zn and Co, which was demonstrated by its ability to suppress hypersensitivity to both metals in yeast mutants, and localizes at the vacuolar membrane (Table 1). *HvMTP1* transcripts are ubiquitously present in barley organs including roots, shoots, and seeds (Podar et al., 2012). *OsMTP1* and *HvMTP1* have high identity (84%) and similarity (90%) at the amino acid level. Based on conservation and phylogenetic relatedness, we may expect a similar function and subcellular localization. Therefore, OsMTP1 is more likely located at the tonoplast.

### Key residues determine metal selectivity of group 1 Zn-CDF MTP transporters

Key positions at structural sites, including single and multiple residue stretches at cytosolic and TMD, contribute to cation selectivity of MTP proteins. Mutations affecting just one of these structural sites can significantly broaden cation specificity. This suggests that the cation is selected throughout the process of ion translocation, possibly with considerable cooperativity (Kawachi et al., 2012).

**Figure 3:**
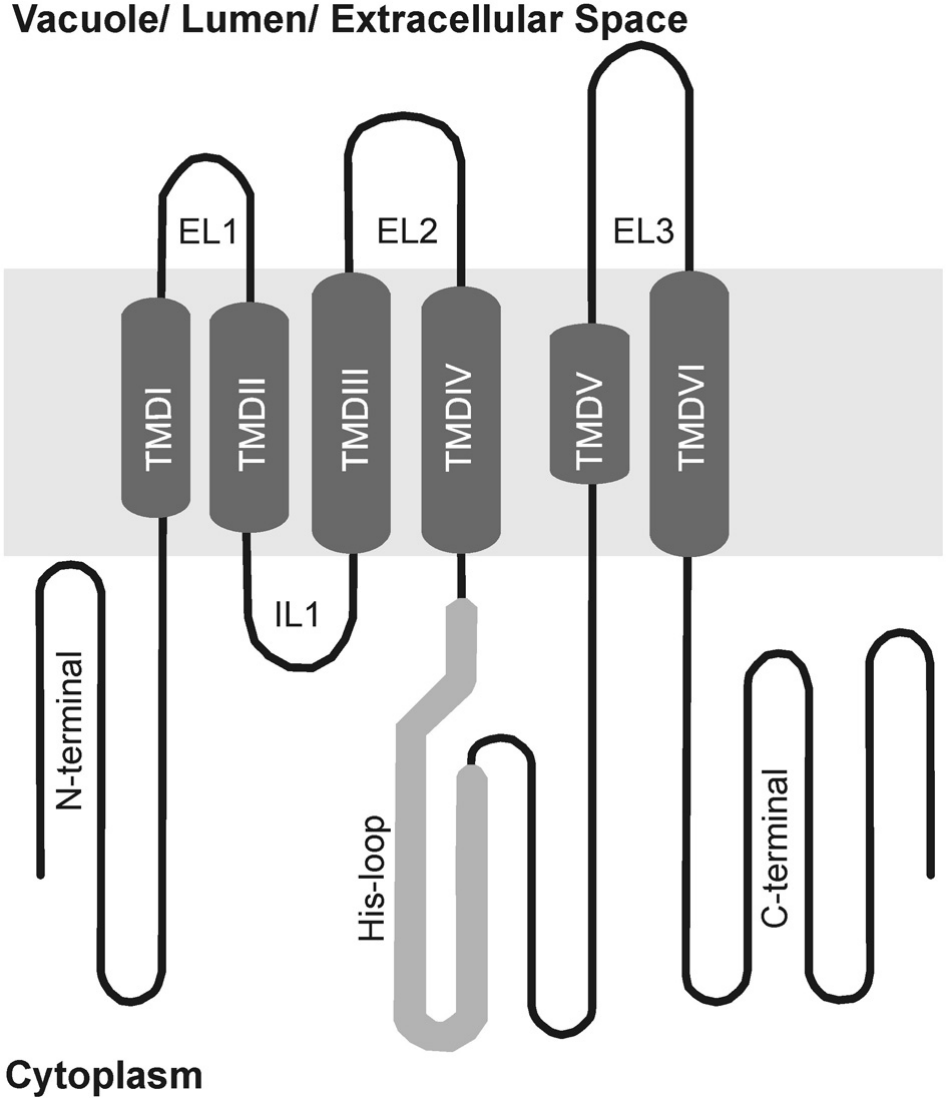
**Schematic membrane topology model of group 1 MTP proteins.** Predicted transmembrane domains (TMDs) in the center (I to VI), cytoplasmic loops below and vacuolar loops above. His-loop, histidine rich loop; IL, intracytosolic loop; EL, extracytosolic loop.

Group 1 MTP proteins contain key polar and charged residues conserved within TMDs I, II, V, and VI, which are likely involved in metal transport of group 1 proteins (Gaither and Eide, 2001; Haney et al., 2005). Besides, a histidine-rich cytoplasmic loop between TMD IV and V (Figure 3) is thought to be vital for transporter specificity, which might act as a chaperone determining the identity of metal ions to be transported and/or as a sensor of cytoplasmic Zn levels (Shingu et al., 2005; Kawachi et al., 2008; Podar et al., 2012). A summary of all MTP1 mutations described in the literature is presented in Table 2. This is the first effort to assemble all information about MTP1 substrate specificity in relation to the presence or absence of key residues.

Removal of the His loop from the NcMTP1 protein from the hyperaccumulator *Noccaea* (formerly *Thlaspi*) *caerulescens* results in a non-functional MTP protein (Kim et al., 2004). However, deletion of the 32 terminal residues from the loop of AtMTP1 confers the ability to complement Co-sensitive phenotypes, while still transporting Zn in yeast (Table 2; Kawachi et al., 2008). MTP proteins from *Nicotiana tabacum* and *N. glauca* complement both Zn and Co-sensitive yeast mutants (Table 1), and the His loop of both species show more His residues than AtMTP1 (Shingu et al., 2005). By swapping the His loops of AtMTP1 and HvMTP1, HvMTP1 lost Co transport activity, and a five-residue (VTVTT) region of the loop was shown to confer specificity to Zn transport when present (Table 2; Podar et al., 2012).

These proteins also contain a Leu-zipper (LZ) motif within TMD VI, a repeating pattern of leucine residues at every seventh position that forms a functional alpha helix (Blaudez et al., 2003). The presence of a LZ motif in MTP proteins was first described in PtdMTP1, from poplar. The authors had discovered that the PtdMTP1 protein forms multimers, possibly stabilized by S–S bridges. Looking for other features possibly involved in protein-protein interactions, they identified a LZ motif in the C-terminal region of the protein (Blaudez et al., 2003). Further experiments demonstrated that the LZ motif is not necessary for oligomer formation, but Leu residues within this motif are required for PtdMTP1 functional activity, possibly mediating subtle interactions that are required for the function of the multimeric complex. Leucine residues within the motif were serially substituted for alanine (Table 2), maintaining the non-polar environment but changing the side-chain. These mutations had increasing negative effects on the Zn-transport ability of PtdMTP1 as they were located closer to the protein's C terminal end (Blaudez et al., 2003). It was suggested that the last Leu residue (Leu-314) of the LZ motif is the most critical one for the function of PtdMTP1 (Blaudez et al., 2003). The LZ motif is conserved in a range of CDF proteins from several species, although some interruptions are common within the motif. The final Leu residue of the LZ was found to be highly conserved among plant CDFs, being probably the most important one for function. Recent works demonstrated that mutations falling at the beginning of the LZ motif have little or no effect on Zn tolerance, although possibly impacting in transport of other metals: AtMTP1 mutation in the residue Leu298 conferred gain of function of Co and Cd transport in yeast experiments and OsMTP1 mutation in the residue L317 enhanced Co tolerance (Table 2; Kawachi et al., 2012; Menguer et al., 2013). In contrast, the last Leu residue of the LZ motif in AtMTP1 (Leu319) is essential for protein function and is predicted to form dimerization contact between two protomers, in analogy to the corresponding residue Leu205 of the broad-specificity divalent cation transporter EcYiiP from *E. coli*, which had its crystal structure previously determined (Table 2; Kawachi et al., 2012).

The signature sequence proposed by Paulsen and Saier (1997) and modified by Montanini et al. (2007) enables predictions regarding uncharacterized MTP family members, as this signature is highly conserved between species and seems to be important for metal transport and specificity. Within the CDF signature, mutations in PtdMTP1 (H89K and H89A; Table 2; Blaudez et al., 2003), AtMTP1 (H90A; Table 2; Kawachi et al., 2012) and OsMTP1 (H90D; Table 2; Menguer et al., 2013) abolish Zn transport, but the effect of the mutation on transport of other metals besides Zn was only tested for OsMTP1, which had no effect on Co transport while enhanced Fe tolerance. In the mammalian metal transporters ZnT5 and ZnT8, mutation in the homologous residues (H451D and H106D, respectively; Hoch et al., 2012) abolished metal selectivity allowing Zn and Cd transport. It was concluded that this motif was important in discriminating between Zn and Cd, and that metal selectivity is tuned by a coordination-based mechanism that raises the thermodynamic barrier to Cd binding (Hoch et al., 2012). Mutations in the residues Thr86 and Leu91 of AtMTP1 conferred tolerance to high levels of Co, whereas changes in Thr86 yielded some level of Cd tolerance, while still transporting Zn (Table 2; Kawachi et al., 2012). In OsMTP1, the mutation L82F appears to confer a gain-of-function: the protein can transport low levels of Zn, with enhanced affinity for Fe, Co, and Mn (Menguer et al., 2013). As AtMTP1 and OsMTP1 can still transport Zn, it appears that these residues contribute to Zn selectivity over other metals.

The AtMTP1 protein contains a total of six cysteine (Cys) residues, all distinct from related microbial proteins but mostly present in plant MTP1 sequences. The mutations C59A or C65A did not confer any substantial modification in yeast tolerance to Zn, whereas mutations C296A and C362A increased it (Table 2). However, mutation of either of the two Cys residues C31A or C36A, which are located within the N-terminal domain, strongly reduced the ability of AtMTP1 to confer Zn tolerance (Table 2). Interestingly, compared to wild type AtMTP1, a mutant carrying an N-terminal deletion of residues 2–12 (Δ2–12) conferred higher Zn tolerance to yeast, but the mutant Δ2–55, which also lacks the Cys residues required for the transport function, did not confer any Zn tolerance at all (Table 2; Kawachi et al., 2012). These results indicate that all or a subset of the 12 N-terminal amino acids of AtMTP1 act negatively on the Zn transport capability, and confirm that the part of the N-terminal domain that includes Cys31 and Cys36 is essential for protein function (Kawachi et al., 2012).

Homology modeling of AtMTP1 based on the 3D structure of *E. coli* YiiP, combined with site-directed mutagenesis and yeast complementation assays, strongly suggests that the active Zn-binding site (site A) of AtMTP1 is formed by His90 and Asp94 in TMD II and His265 and Asp269 in TMD V, with conserved positions of Zn-binding residues (Table 2). These key residues of the Zn-binding site are highly conserved in numerous other CDF family proteins (Kawachi et al., 2012). The corresponding aspartate residue in *S. cerevisiae* Zrc1, when mutated to alanine, results in loss of Zn transport activity, suggesting functional conservation of the corresponding residues. Consistent with this finding, it was shown that mutations in four critical residues in TMD II (Leu33, Phe40, Asn44, and Ala52) affected metal specificity in Zrc1 (Lin et al., 2009).

Some other residues such as Glu72, Asp87, Glu124, Asn173, and Asp293 are also important for the Zn transport function of AtMTP1 (Table 2). Based on 3-D AtMTP1 modeling, it was suggested that Glu72, Asp87, and Asn173 are located close to the active Zn-binding site; Asp293 is located in the opening of the cavity to the vacuole; and Glu124 is located at the entrance of the cavity facing the cytosol (Kawachi et al., 2012). Although these residues are not necessary for Zn binding, they may be involved in translocation of Zn and/or protons through the membrane (Kawachi et al., 2012).

## MTPs from Group 8 and 9 of the Mn-CDF cluster

According to phylogenetic analyses, groups 8 and 9 of the CDF superfamily have an ancient origin, containing both prokaryote and eukaryote sequences. Products of a possible duplication event, these two distinct groups have been functionally characterized as Mn transporters (Gustin et al., 2011). The protein ShMTP8 (originally ShMTP1, name changed to maintain consistency with *Arabidopsis* nomenclature), encoded by the tropical Mn hyperaccumulating legume *Stylosanthes hamata*, was the first characterized transporter from the Mn-CDF group in plants. Through the expression of a cDNA library prepared from *S. hamata* in the yeast *S. cerevisiae*, Delhaize et al. (2003) identified four cDNAs encoding membrane-bound proteins of the CDF superfamily which confer Mn tolerance to yeast. One of these cDNAs, *ShMTP8*, confers Mn tolerance in yeast by sequestration of Mn^2+^ to an internal organelle, instead of causing Mn^2+^ efflux to the media. Such Mn transport must function as an H^+^:Mn^2+^ antiporter, due to the requirement of an active V-type H^+^-ATPase for effective Mn tolerance. In yeast, the protein fused with GFP appeared to localize at the endoplasmic reticulum, but such localization is inconclusive due to the lack of an ER marker. ShMTP8 over-expression in plants (*Arabidopsis* and tobacco) also conferred Mn^2+^ tolerance; however, it was localized specifically at the tonoplast (Figure 2), strongly suggesting that Mn^2+^ sequestration to the vacuole is the mechanism conferring Mn^2+^ tolerance (Delhaize et al., 2003).

The high similarity of ShMTP8 with AtMTP8 to 11 suggests that these proteins may have similar functions. Of the four tested genes, *AtMTP11* showed the highest expression levels in whole seedlings of *Arabidopsis* grown under different Mn^2+^ supplies. As expected, when expressed in yeast cells, AtMTP11 conferred enhanced tolerance to Mn^2+^ (and to a lesser extent to Cu^2+^), but no increased tolerance to a range of other metals (Zn^2+^, Co^2+^, or Ni^2+^) was detected. Using yeast microsomal membrane vesicles, it was shown that AtMTP11 also confers Mn^2+^-dependent proton-transport activity (Delhaize et al., 2007). In contrast to ShMTP8, AtMTP11 was not located at the vacuolar membrane, but was found in pre-vacuolar compartments or trans-Golgi (Figure 2; Delhaize et al., 2007; Peiter et al., 2007). It was suggested that the Mn tolerance conferred by AtMTP11 probably relies on vesicular trafficking and exocytosis of excess Mn^2+^ (Peiter et al., 2007), which is corroborated by the increased accumulation of Mn in leaves of *Arabidopsis* mutant plants with disrupted *AtMTP11* gene (*mtp11*). The same *Arabidopsis* mutant plants are hypersensitive to elevated levels of Mn, whereas over-expressing plants are hypertolerant (Delhaize et al., 2007; Peiter et al., 2007). Interestingly, two homologous genes from poplar, PtMTP11.1 and PtMTP11.2, are also targeted to a Golgi-like compartment and are able to complement *mtp11 Arabidopsis* mutant plants, suggesting that poplar and *Arabidopsis* MTP11 proteins have similar functions (Peiter et al., 2007). As previously stated by Gustin et al. (2011), it is clear that at least some of the CDF members from MTP groups 8 and 9 are important to Mn homeostasis, and the early bifurcation and further expansion of these groups represent the adaptive strategies that each plant species has developed to maintain Mn homeostasis and to deal with Mn toxicity.

## Other MTPs from the CDF superfamily (Groups 5, 6, 7, and 12)

Similarly to other groups from the CDF superfamily, groups 5, 6, 7, and 12 have an ancient origin, including CDF sequences from diverse prokaryote and eukaryote organisms (Gustin et al., 2011). However, in contrast to groups 1, 8 and 9, these four groups have maintained only one copy of each sequence within genomes of the analyzed plants. Although the existence of most of the members in these groups is supported by cDNAs or ESTs sequences, the only functional evidence for these groups comes from a high throughput ionomic study which investigated several *A. thaliana* accessions and mutant lines (Baxter et al., 2007). Two mutant lines screened (*mtp5* and *mtp6*, members of groups 5 and 6, respectively) showed alterations in the ionome of mutant leaves. Repeatable alterations in multiple ions in the *mtp5* mutant leaves included reduced levels of Mo, Mn, and Mg and increased levels of K and Zn. The *mtp6* mutant showed consistent diverse alterations in the ionome with reduced levels of Mg, Mo, and Ca and increased levels of Na, K, Mn, and Cd. Therefore, it was suggested that both AtMTP5 and AtMTP6 have important roles in regulating ion concentrations in *A. thaliana* under normal conditions (Gustin et al., 2011). Although members of the group 6 have been included in the Zn/Fe-CDF group (Montanini et al., 2007), no functional data is available regarding the substrate specificity of these transport proteins in plants. If functional characterization of group 6 members confirms the hypothesis of Zn/Fe transport, these sequences could be used as putative targets in plant biofortification strategies.

## MTP proteins in metal hyperaccumulator species

### Role of MTP1 in the hypertolerance trait of hyperaccumulators

Metal hypertolerant/hyperaccumulator plants are able to accumulate from one to four orders of magnitude higher metal concentrations in their above-ground biomass compared to other plants growing in the same environment. Metals accumulated include Zn, Mn, Cd, Co, Ni, Cu, selenium (Se), arsenic (As), lead (Pb), antimony (Sb), and thallium (Tl) (Baker and Brooks, 1989; Reeves and Baker, 2000; Krämer, 2010). These traits were reported in ~500 taxa so far, and are especially common in the Brassicaceae family (Krämer, 2010). The study of hypertolerance/hyperaccumulation traits is attractive in Brassicaceae because of the high similarity of “model” hyperaccumulators *Arabidopsis* (former *Cardaminopsis*) *halleri* and *Noccaea* (former *Thlaspi*) *caerulescens* with the model species *A. thaliana*, allowing use of the large number of tools available to the *Arabidopsis* community in cross-species comparisons. Due to that, we focused on the role of MTP proteins in hypertolerance/hyperaccumulation in these species.

*A. halleri* is a Zn and Cd hypertolerant/hyperaccumulator (Krämer, 2010) that shares 94% nucleotide identity within coding regions with *A. thaliana* (Koch et al., 1999, 2000; Clauss and Koch, 2006). *N. caerulescens* has diverged earlier, sharing 88% nucleotide identity (Peer et al., 2003, 2006). *A. halleri* and *N. caerulescens* are constitutively Zn-tolerant and Zn hyperaccumulators, with intraspecific variation for Cd and/or Ni (Milner and Kochian, 2008; Krämer, 2010). Although metal accumulation evolved independently in *A. halleri* and *N. caerulescens* (Krämer, 2010), they share a common set of alterations, indicating that a small number of constraints need to be changed for metal hyperaccumulation/hypertolerance traits to emerge (Hanikenne and Nouet, 2011).

Hyperaccumulators change metal partitioning between roots and shoots. In non-hyperaccumulators, metal shoot-to-root ratio is below unity, whereas in hyperaccumulators it is generally above (Baker et al., 1994; Talke et al., 2006; Krämer, 2010). Both hyperaccumulation and hypertolerance result from changes in physiological processes, namely: (1) root metal uptake; (2) enhanced xylem loading for root-to-shoot translocation; and (3) enhanced metal sequestration and detoxification in shoots (Hanikenne and Nouet, 2011). Studies in *A. halleri* and *N. caerulescens* indicate that ZIP influx transporters are probably involved in metal uptake (Talke et al., 2006; van de Mortel et al., 2006; Weber et al., 2006) whereas the presence of multiple copies of HMA4 efflux transporter genes in both hyperaccumulators is responsible for increased xylem loading and subsequent root-to-shoot translocation (Hanikenne et al., 2008; O'Lochlainn et al., 2011). In shoots, a key protein for metal sequestration and detoxification is the vacuolar protein MTP1 (Figure 2; Dräger et al., 2004; Gustin et al., 2009).

Root and shoot transcriptomic comparisons indicated that *AhMTP1* transcripts are constitutively higher in *A. halleri* than in *A. thaliana*, especially in leaves (Becher et al., 2004; Dräger et al., 2004; Talke et al., 2006). AhMTP1 is a vacuolar protein that is able to complement the Zn-sensitive phenotype of the *zrc1cot1* mutant yeast (Table 1; Dräger et al., 2004). Surprisingly, three copies of the *AhMTP1 loci* were first identified, explaining the observed high transcript levels. Segregant populations of *A. halleri* × *A. lyrata* crosses showed that only two out of the three identified *A. halleri MTP1 loci* co-segregate with Zn tolerance (Dräger et al., 2004). This was later confirmed by another study showing that the *A. halleri* genome has 4–5 *MTP1* paralogs, located at four *loci*, with one copy not fixed in the population analyzed (*AhMTP1-D*, see below; Shahzad et al., 2010). *AhMTP1-A1* and *AhMTP1-A2* are duplicated *in tandem* and thus linked, whereas *AhMTP1-B*, *AhMTP1-C*, and *AhMTP1-D* are segmentally duplicated (Shahzad et al., 2010). *AhMTP1-A1*/*AhMTP1-A2* and *AhMTP1-B loci* are the copies associated with high accumulation of MTP1 transcripts in shoots, up-regulation upon high Zn concentrations in roots, and Zn hypertolerance (Dräger et al., 2004; Shahzad et al., 2010). Interestingly, although all five copies were able to confer Zn-tolerance to *zrc1cot1* mutant yeast, AhMTP1-B was less competent than AhMTP1-C and AhMTP1-D (Table 1; Shahzad et al., 2010). These results indicated that although duplication of MTP1 loci could be the basis of Zn tolerance in *A. halleri*, the five MTP genes seem to be undergoing distinct evolutionary fates (Shahzad et al., 2010).

An *N. caerulescens* MTP1 protein was cloned by homology with *AtMTP1* (van der Zaal et al., 1999) and named *NcZTP1* (previously *TcZTP1*, Assunção et al., 2001). *NcZTP1*, renamed *NcMTP1*, was shown to be highly expressed in leaves compared to homologous genes in its closest non-hyperaccumulator relative, *Thlaspi arvense*, and in *A. thaliana* (Assunção et al., 2001). *NgMTP1*, an ortholog from another Zn hyperaccumulator, *Noccaea* (former *Thlaspi*) *goesingense*, was also highly expressed in shoots compared to non-hyperaccumulators *A. thaliana*, *T. arvense*, and *Brassica juncea* (Persans et al., 2001). NgMTP1 was shown to complement Zn, Cd, and Co-sensitivity in yeast (Table 1; Persans et al., 2001; Kim et al., 2004). At first, transient expression of a GFP-tagged version of NgMTP1 in *A. thaliana* protoplasts showed plasma membrane localization, which is unusual for MTP1 homologs (Kim et al., 2004). In yeast, NgMTP1 was localized at both the plasma membrane and the vacuolar membrane, acting in Zn efflux from the cell as well as in vacuolar storage (Kim et al., 2004). However, a later work using NgMTP1-specific antibody and membrane fractionation demonstrated that NgMTP1 is vacuolar. Both the native protein in *N. goesingense* and the protein heterologously expressed in *A. thaliana* were localized at the tonoplast, in agreement with the localization of other MTP1 homologs (Gustin et al., 2009; Table 1).

*NgMTP1* expression under control of the 35S promoter in *A. thaliana* led to increased Zn concentration in roots, but decreased in shoots (Gustin et al., 2009). Reciprocal grafting of wild type and 35S::*NgMTP1 A. thaliana* lines showed that shoot-specific NgMTP1 expression leads to Zn accumulation in leaves, as well as up-regulation of *AtZIP4* and *AtZIP5* in shoots and *AtZIP3* and *AtZIP9* in roots, characteristic of Zn-deficiency response (Grotz et al., 1998; Wintz et al., 2002, 2003; van de Mortel et al., 2006; Talke et al., 2006; Gustin et al., 2009). Importantly, it was demonstrated that metal tolerance is dependent on NgMTP1 expression in shoots (Gustin et al., 2009). These results were confirmed in reciprocal grafting experiments of *N. caerulescens* with the non-hyperaccumulator *Noccaea perfoliatum*, which demonstrated that hyperaccumulation is a root-driven phenotype, whereas hypertolerance is shoot-driven (de Guimarães et al., 2009). Considering that *NgMTP1* transcripts are more abundant in shoots of *N. goesingense* than in non-hyperaccumulators (Persans et al., 2001), it is clearly established that shoot MTP1 expression has a pivotal role in the Zn hypertolerance trait.

*MTP1* genes from the Zn/Cd hyperaccumulating and non-hyperaccumulating ecotypes of *Sedum alfredii* were both able to suppress Zn hypersensitivity in the *zrc1* yeast mutant (Table 1), induced Zn accumulation and were localized at the tonoplasts of onion cells (Zhang et al., 2011). As described for *A. halleri* (Dräger et al., 2004), the amount of *MTP1* transcripts of the hyperaccumulating ecotype is higher in shoots than in roots, a difference not observed in the non-hyperaccumulator ecotype. Upon Zn or Cd excess treatment, *MTP1* transcripts accumulate in shoots of the hyperaccumulator and in roots of the non-hyperaccumulator ecotype (Zhang et al., 2011). These results indicate that, as in other hyperaccumulator species, *S. alfredii* hypertolerance probably relies on increased expression of MTP1 transporters in shoots.

The mesophyll cells are the major site of metal accumulation in *A. halleri* (Küpper et al., 2000; Sarret et al., 2009), whereas the epidermal cells show higher Zn concentrations in *N. caerulescens* (Küpper et al., 1999; Schneider et al., 2012). Unexpectedly, *in situ* hybridization and proteomics approaches showed that *N. caerulescens* MTP1 is not differentially expressed, but rather equally distributed in epidermal and mesophyll cells (Schneider et al., 2012). Protein fragments homologous to plasma membrane influx transporters from the ZIP family were enriched in the epidermal fraction, which would explain the differential accumulation observed (Küpper and Kochian, 2010; Schneider et al., 2012). Therefore, it is suggested cell type-specific expression of MTP1 is not driving differential accumulation of metals, which would be based on other proteins such as influx transporters.

A model explaining the role of MTP1 in the hyperaccumulation phenotype has been proposed. High root-to-shoot translocation of Zn is achieved by HMA4, whereas metal detoxification occurs at the vacuoles of leaf cells by MTP1 transporter proteins (Gustin et al., 2009; Hanikenne and Nouet, 2011). In this model, HMA4 would be necessary for hyperaccumulation, whereas MTP1 would be necessary for hypertolerance. In agreement with the model, *A. thaliana* plants expressing *AhHMA4p*::*AhHMA4* constructs hyperaccumulated Zn in shoots, but were not tolerant to high Zn concentrations (Hanikenne et al., 2008). Plants expressing *NgMTP1* in shoots were hypertolerant to high Zn but did not accumulate metals to the same extent as hyperaccumulators (Gustin et al., 2009). It was also shown that a Zn accumulation QTL co-localized with *HMA4* (Frérot et al., 2010), and two Zn tolerance QTLs were co-localized with the *MTP1 loci* and another one with *HMA4* in *A. halleri* (Willems et al., 2007). Still, it is possible that MTP1-dependent increase of sink strength in shoots might have a minor, secondary effect on hyperaccumulation, depleting cytoplasmic pools of Zn in leaves, and increasing Zn demand (Becher et al., 2004; Gustin et al., 2009), as suggested for HMA4 root-to-shoot metal translocation activity (Talke et al., 2006; Hanikenne et al., 2008; Hanikenne and Nouet, 2011). The full understanding of molecular mechanisms involved in hyperaccumulation/hypertolerance will be directly applicable to phytomining and phytoremediation, as well as crop nutrient use efficiency and biofortification (Clemens et al., 2002).

## MTP proteins as tools for biofortification

The only source of metal micronutrients for humans is the diet, and in developing countries plants are the main source of these metals. Consequently, the presence of minerals and the maintenance of their homeostasis within the edible tissues of plants are of great importance for human nutrition. Biofortification of grains has been considered a promising strategy to improve human nutrition, and expression of genes encoding metal transporters, alone or in combination with other genes, has been suggested as a useful approach in this direction (Palmgren et al., 2008; Sperotto et al., 2012).

During the grain-filling stage, plants remobilize and transport nutrients distributed throughout the vegetative source organs into seeds [reviewed by Waters and Sankaran (2011)]. Micronutrients such as Fe, Zn, Cu, and Mn are mainly localized in the seed aleurone layer, where phytic acid (main form of Pi storage in seeds) acts as a strong chelator of metal cations and bind them to form phytate, a salt of inositol phosphate (Raboy, 2009). In developing rice grain, Zn stays near the aleurone layer and moves more deeply into the inner endosperm than other micronutrients (Iwai et al., 2012). In barley, *MTP1* is expressed in all tissues of the grain (transfer cells, aleurone, endosperm, and embryo) with the lowest expression in transfer cells and the highest in aleurone and embryo, where Zn accumulates at higher levels (Tauris et al., 2009). The biotechnological potential of *MTP1* for Zn biofortification purposes has already been highlighted (Palmgren et al., 2008; Anuradha et al., 2012; Podar et al., 2012). In future experiments, an interesting approach would be endosperm-specific overexpression of *MTP1*, which could lead to enhanced accumulation of Zn in edible tissues. In a complementary strategy, specific silencing of MTP proteins responsible for metal accumulation in leaf vacuoles could increase the amount of available minerals to be transported to grains.

Mutations in amino acid residues within the MTP1 protein may also be important for altering the selectivity of transport for biofortification purposes. Indeed, one of the major challenges in biofortification efforts is to avoid increasing concentrations of undesirable toxic metals in edible plant organs. Micronutrient transporters often have broad substrate specificity: an example is the Arabidopsis Fe^2+^ transporter AtIRT1, which is also able to transport Mn^2+^, Zn^2+^, Cd^2+^, Co^2+^, and Ni^+2^ (Eide et al., 1996; Korshunova et al., 1999; Vert et al., 2002; Nishida et al., 2011). Because of this, plants over-expressing AtIRT1 have increased accumulation of Fe, but also of other toxic metals, and efforts have been made to alter protein selectivity (Rogers et al., 2000; Barberon et al., 2011). Thus, the determination of amino acid residues that are important for metal specificity is useful in two ways: to generate transporters that would select only beneficial ions such as Fe and Zn, which would be used to increase the concentrations of these metals in edible parts of plants; and to generate transporters that specifically transport toxic ions such as Cd, trapping them inside the vacuoles or other compartments of non-edible plant organs. Any of these approaches would need careful choice of specific promoters.

Recent studies with the Arabidopsis MTP1 protein failed to discover a single substitution that entirely shifts the substrate specificity of AtMTP1 from Zn (Kawachi et al., 2012; Podar et al., 2012), suggesting that higher organisms regulate transport tightly by holding more than one residue responsible for substrate specificity (Podar et al., 2012). However, the H90D mutation in rice OsMTP1 abolishes Zn transport and enhances Fe tolerance (Menguer et al., 2013). Fortunately, some mutations have the capacity to allow MTP1 to transport Fe along with Zn, making it a good candidate for biofortification approaches (Kawachi et al., 2012; Podar et al., 2012). Fe and Zn deficiency are the most common mineral nutritional disorders in humans, and simultaneous increases in Fe and Zn concentrations would be highly beneficial.

Since studies with MTP proteins from hyperaccumulators demonstrated that high copy number and enhanced expression are more likely explanations for hypertolerant/hyperaccumulator phenotypes (Dräger et al., 2004; Shahzad et al., 2010), it would seem that genes from metal hyperaccumulator species have no special features to distinguish them from those of ordinary plants, and that the biotechnological approach aiming at biofortified crops would be similar when using genes obtained from either group of plants. However, the ShMTP8 protein seems to be more efficient in Mn^2+^ transport than AtMTP11 (Delhaize et al., 2003, 2007). This improved efficiency in a protein from a hyperaccumulator species indicates that hyperaccumulators are potential sources of useful new genes for biofortification.

## Conclusions and further directions

There seems to be enough variation within the MTP family of transporters to provide a wide range of candidate genes to be used in specific biotechnological applications in plants, including biofortification, phytoremediation, and improved efficiency in the use of nutrients (avoiding deficiency and toxicity). Successful strategies will depend on the amount of information available about the specific function of each of these proteins, as well as on the appropriate combinations of genes and promoters. As an example, Masuda et al. (2012) recently showed that basic knowledge on Fe homeostasis-related proteins, such as the Fe(II)-NA transporter OsYSL2, the NA synthase OsNAS2 and the Fe-binding protein Ferritin, combined with the right choice of promoters, can lead to increased Fe concentrations in the rice endosperm.

Most certainly, the exploitation of specific promoters should be pursued in strategies for improving genotypes for particular biotechnological purposes. Although useful in basic studies of gene function, constitutive over-expression changes metal homeostasis in all organs, and often affects uptake and distribution of more than one element. Knowledge about the expression patterns of diverse MTP genes may also provide useful promoters for expression of other transporters, with specific substrate preferences, in a tissue- or cell layer-targeted fashion.

Manipulation of CDF substrate specificity may also be a promising avenue. The description of the 3D structure of a bacterial CDF transporter, YiiP (Lu and Fu, 2007), presents an opportunity to apply molecular modeling to spatially understand why certain residues are determinants of metal selectivity in other members of the family. This approach was successfully applied to AtMTP1 (Kawachi et al., 2012) and could be used more widely in the future as the basis for tailoring the substrate specificity of CDFs for targeting particular metals. Although MTP proteins are able to transport metals other than Zn, site-directed mutagenesis can be employed to change specificity into a broader or narrower range, depending on the desired outcome.

Most MTP proteins have not yet been functionally characterized, and the subcellular localization of some of those characterized is still a matter of controversy. Therefore, further work is required before the roles of all family members are fully understood. Substrate specificity, sub-cellular localization, and expression patterns (in different tissues, developmental stages, and in response to a wide range of treatments) are crucial information to be sought. Even in Arabidopsis, there are MTP proteins which are not functionally characterized. In this species, and in others that have similar resources, characterization of insertion mutants, and over-expressing lines should help clarify the physiological relevance of MTP genes. Additionally, ionomic studies used with these plants will provide the opportunity of a wider understanding of the role of MTPs and their influence on the relationship between different elements at different stages of growth and development and under a variety of environmental conditions (Williams and Salt, 2009). The more we learn about MTP proteins, the greater the chances of designing successful biofortification programs that make use of their appropriate and rational manipulation.

### Conflict of interest statement

The authors declare that the research was conducted in the absence of any commercial or financial relationships that could be construed as a potential conflict of interest.
